# Psychopathological and sociodemographic features in treatment-resistant unipolar depression versus bipolar depression: a comparative study

**DOI:** 10.1186/s12888-018-1641-y

**Published:** 2018-03-16

**Authors:** Nicolas A. Nuñez, Stefano Comai, Eduard Dumitrescu, Maykel F. Ghabrash, John Tabaka, Marie Saint-Laurent, Stephen Vida, Theodore Kolivakis, Allan Fielding, Nancy Low, Pablo Cervantes, Linda Booij, Gabriella Gobbi

**Affiliations:** 1 Department of Psychiatry, Neurobiological Psychiatry Unit, McGill University Health Center (MUHC), McGill University, Room 220, 1033 Pine Avenue West,, Montreal, QC Canada; 2 Department of Psychiatry, Mood Disorder Clinic, McGill University Health Center, McGill University, Montreal, QC Canada; 3 Division of Neuroscience, San Raffaele Scientific Institute and Vita-Salute University, Milan, Italy; 4 Department of Psychology, Concordia University and Sainte-Justine Hospital Research Center, University of Montréal, Montréal, QC Canada

**Keywords:** Treatment-resistant depression, Bipolar depression, Psychopathology, Affective disorders, Bipolar spectrum

## Abstract

**Background:**

Some authors have hypothesized that Treatment-Resistant Unipolar Depression (TRD-UP) should be considered within the bipolar spectrum disorders and that hidden bipolarity may be a risk factor for TRD-UP. However, there are neither studies comparing clinical and sociodemographic data of patients with TRD-UP versus Bipolar (BP) disorders nor are there any examining differences versus Bipolar type I (BP-I) and Bipolar type II (BP-II).

**Methods:**

Charts analysis was conducted on 194 patients followed at the Mood Disorders Clinic of the McGill University Health Center. Sociodemographic, clinical features and depression scales were collected from patients meeting DSM-IV criteria for TRD-UP (*n* = 100) and BP (*n* = 94). Binary logistic regression analysis was conducted to examine clinical predictors independently associated with the two disorders.

**Results:**

Compared to BP, TRD-UP patients exhibited greater severity of depression, prevalence of anxiety and panic disorders, melancholic features, Cluster-C personality disorders, later onset of depression and fewer hospitalizations. Binary logistic regression indicated that higher comorbidity with anxiety disorders, higher depression scale scores and lower global assessment of functioning (GAF) scores, and lower number of hospitalizations and psychotherapies differentiated TRD-UP from BP patients. We also found that the rate of unemployment and the number of hospitalizations for depression was higher in BP-I than in BP-II, while the rate of suicide attempts was lower in BP-I than in BP-II depressed patients.

**Conclusions:**

These results suggest that TRD-UP constitutes a distinct psychopathological condition and not necessarily a prodromal state of BP depression.

## Background

Depressive disorders are considered as one of the major worldwide public health burdens [1]. Treatment-Resistant Unipolar Depression (TRD-UP) continues to be a clinical challenge due to its heterogeneous presentation with an impact on functional impairment, declined autonomy, and poor cognitive functioning [2]. Although advances have been made to improve our psychiatric diagnostic classification systems, many intermediate phenotypes have not been accurately diagnosed and proposed predictors of treatment outcomes in depression seem controversial with remission rates remaining unchanged [3].

Over the years there has been several definitions proposed as to adequately define TRD-UP [4, 5]. TRD-UP can be defined either as the failure to respond to the first antidepressant (AD) trial [6] or two or more AD trials [7] of different classes of AD [8]. It has been described that up to 15% of patients treated for depression will fall into this category [9] and according to the Sequenced Treatment Alternatives to Relieve Depression (STAR*D) study more than 50% of depressed patients do not respond to their first AD trial [10]. However, there is currently no universal definition of TRD-UP and controversies surrounding its prevalence rates, definitions and treatment outcomes remain ambiguous [11, 12].

A number of clinical and demographic characteristics have been found to be associated with TRD-UP. These include comorbidity with anxiety panic disorder, social phobia, personality disorder, suicidal risk, melancholia, number of hospitalizations, recurrent episodes, early age of onset, total number of unresponsive treatments to antidepressants received during a lifetime [13] as well as severity of depression and having a first degree relative with an affective disorder [14].

It has been proposed that TRD-UP can be considered a “prodromal phase” of Bipolar disorder (BP) included in the bipolar spectrum disorders and a sub-threshold bipolarity or hidden bipolarity as a risk factor for TRD-UP [15]. This hypothesis has been confirmed by a recent systematic review examining possible risk factors for treatment resistance in unipolar major depression, in which, among others, the presence of a non-diagnosed bipolarity was found to be an independent risk factor for treatment resistance [16].

The diagnostic distinction between TRD-UP and BP is of paramount importance for the treatment and prognosis of depression. While TRD-UP must be treated with a combination of different classes of antidepressants (AD) or with second-generation antipsychotic (SGA) augmentation strategies [17] in BP depression, AD must be carefully used and monitored considering that they may induce a switch in mania, hypomania or symptoms such as psychomotor activation, insomnia or irritability [18, 19]. Unfortunately, it is still a challenge to accurately predict if a TRD-UP could be a masked form of BP depression.

Other studies examining the differences between UP (non-TRD) and BP depression revealed that the prevalence of characteristics such as age of onset was lower but that the total number of depressive episodes as well as the presence of a family history of depression was higher in BP than in UP depression [20–23]. Therefore, while some different characteristics between UP and BP depression have been well characterized, the different demographic, social, and clinical characteristics associated with TRD-UP versus BP depression have not yet been studied, although this early differential diagnosis is pivotal for improving diagnostic and therapeutic outcomes.

In this retrospective and observational cross-sectional chart-review study, we have examined clinical and demographic characteristics mostly associated with the diagnosis of TRD-UP or BP that have been previously described in the literature as risk factors or predictors for these disorders [14, 16, 24]. The goal was to find clinical and socio-demographical characteristics to assist clinicians to better differentiate between TRD-UP from depression as part of the bipolar spectrum disorders. As a secondary goal, given the subtypes of the bipolar spectrum, we investigated whether there were clinical and socio-demographical characteristics that differed between Bipolar Type I (BP-I) and Type II (BP-II) disorders and between them and TRD-UP.

## Methods

The study was approved by the Institutional Review Board of McGill University (13–375-PSY) and was conducted in accordance with the Declaration of Helsinki and ICH Good Clinical Practice. Chart reviews were collected in the Patient Registry at the Mood Disorders Clinic (MDC) of the McGill University Health Center (MUHC). The Patient Registry at the MDC is a research database where uniform data is collected on all UP and BP disorder patients who are treated and followed at the clinic for more than 2 years (mean 7.5 years). Being a chart reviews study, the informed consent was not required.

### Patients

Patients meeting the DSM-IV criteria for a major depressive episode (MDE) within a UP or BP diagnosis were included in the study [25]. The medical charts of 194 outpatients between the ages of 19–75, with a MDE and meeting DSM-IV criteria for TRD-UP (*n* = 100) and BP (*n* = 94) were reviewed. Among the BP patients, 52 were diagnosed with BP-I and 42 with BP-II. Patients with UP major depressive disorder met criteria for TRD-UP by failing at least two adequate trials with different AD in mono or combination therapy at the adequate dose and for at least three weeks [7].

The patient’s diagnoses were ascertained by the Structured Clinical Interview for Diagnosis (SCID) [26] which was conducted by psychiatrists or professionals who received a training in SCID. The Maudsley Staging Method (MSM) was used to establish the severity of the TRD patients [27]. In addition, the Young Mania Rating Scale (YMRS) [28] was used to evaluate whether patients currently displayed acute hypomanic or manic symptoms and if they did not meet the criteria for a mixed episode of depression at the time of the assessment.

The inclusion criteria included patients with a diagnosis of a MDE ranging from mild to severe intensity measured by a score greater than 20 on the Montgomery–Asberg Depression Rating Scale (MADRS) and a score greater than 13 on the Hamilton-Rating Scale for Depression (HAM-D17) [29]. The duration of the current episode had to be greater than two months. Patients with a mixed episode, currently in a manic episode or with the presence of a neurological/developmental disorder and/or a mood disorder secondary to a medical condition were excluded.

Patients were selected during the phase of depression, before the administration of a stable and effective psychopharmacological treatment (treatment not changed by the psychiatrist for at least three months).

Pharmacological treatment at time of evaluation was as follows: for the TRD-UP group, 38 patients were treated with AD mono/combination therapy and 62 patients were treated with an augmentation strategy that included AD in combination with SGA (*n* = 49) or mood stabilizers (MS) (*n* = 13). In the BP group, patients were treated with MS in combination with SGA (*n* = 30), AD in combination with SGA and MS (*n* = 23), AD plus MS (*n* = 21), AD plus SGA (*n* = 10), MS monotherapy (*n* = 5), and SGA monotherapy (n = 5).

### Clinical evaluation

A retrospective chart analysis was performed by two raters and clinical features were evaluated in the two groups. The following scales were considered for depression severity: Montgomery–Asberg Depression Rating Scale (MADRS) [30]; Clinical Global Impression-Severity of Illness (CGI-S) [31]; Quick Inventory of Depressive Symptomatology (QIDS-C16) [32] and Hamilton-Rating Scale for Depression (HAMD-17) [33].

The following patient socio-demographic information was obtained from the MDC Patient Registry: age, ethnicity, gender, marital status, employment, level of education and living arrangement as well as previous psychiatric diagnosis including Attention Deficit-Hyperactivity disorder (ADHD), alcohol or substance abuse, anxiety disorders, sleep disorders and eating disorders. Information was also collected on family history of affective disorders, age of first psychiatric consultation, age of first depressive episode and the number of depressive episodes, age of first manic episode and number of manic episodes, age of first hypomanic episode and number of hypomanic episodes. Data was also collected on the history of psychotherapy, electrical or neurological therapy, use of psychiatric services, general medical history, and number of previous suicide attempts, major depression with psychotic features, axis II, III and IV DSM-IV-TR [25] pathology, previous and current pharmacotherapy.

Patients were also assessed having depressive melancholic features and depressive atypical symptoms as defined by DSM-IV criteria [25]. Patients within the TRD-UP group had a level of resistant depression of moderate intensity according to the MSM (Mean ± SEM, 9.7 ± 0.2) and patients with BP disorders did not display current manic episodes defined by the YMRS scale (Mean ± SEM: 3.0 ± 0.7).

### Reliability and inter-rater agreement for psychometric scales

A reliability analysis was performed to determine the internal consistency by means of Cronbach’s alpha. Overall, we reached an acceptable reliability for all the scales (MADRS: α = 0.91; HAMD-17: α = 0.82; QIDS-C16: α = 0.77).

Inter-rater reliability was performed on a sample of 140 patients. Patients were assessed by three raters (two psychiatrists and a General Practitioner). We found moderate to good agreement (Cohen’s kappa range: 0.58–0.85) [34]) (MADRS: 0.60; HAMD-17: 0.58; QIDS-C16: 0.61; CGI-S: 0.72; CGI-Global Improvement: 0.85) across all scales.

### Statistical analysis

Statistical analysis was conducted using the Statistical Package for the Social Sciences (SPSS-23; SPSS Inc., Chicago, IL, USA). Data are presented as mean ± standard deviation (SD). Inter-rater reliability for individual scales was calculated using Cohen’s kappa [35].

As an initial step, we considered 40 variables that were compared between TRD-UP and BP by Student’s *t* test for continuous variables or by Pearson’s chi-square (χ^2^) test for categorical variables. Then, using a binary logistic regression analysis we examined which variables were specific predictors of the two affective disorders. Given the high number of variables under investigation, and to balance risk for type I and type II errors, we choose to include in the binary logistic model only those variables that in the initial step were significantly different between the two groups at an alpha level of 0.01. Moreover, we excluded from the model those variables for which few individuals (*n* ≤ 5) were affected by a specific disorder in at least one of the two groups. Predictors reaching *p* < 0.01 were considered significant.

As final step, we investigated for possible differences in the clinical and demographic characteristics of TRD-UP, BP-I and BP-II patients. To examine possible differences for categorical variables, we first tested at an alpha level of 0.05 the overall 3 × 2 matrix containing all the three affective disorders. For statistically significant variables, we subsequently conducted multiple 2 × 2 cross tabulations using Pearson’s chi-square (χ^2^) test. For comparisons concerning continuous variables, we used the analysis of variance (ANOVA) followed by Bonferroni post-hoc test for multiple comparisons.

## Results

### Sociodemographic characteristics

The mean age (± SD) of the total sample was 43.6 (±14.1) years with 58.3% of the participants consisting of females (*n* = 116) and 39.2% (*n* = 78) of males. At the time of evaluation, 68.3% of the patients were unemployed (*n* = 136) and 40.1% had a single status (*n* = 81). Table 1 summarizes and compares the sociodemographic and clinical features of TRD-UP and BP patients.

**Table 1:** Socio-demographic and clinical characteristics of patients with TRD-UP and BP disorder (*N* = 194)

	TRD-UP (*n* = 100)	BP (*n* = 94)	Statistics
Age (Years) (Mean ± SD)	46.5 ± 13.3	40.6 ± 14.3	*t* = 2.97_,_ ***p*** **= 0.003**
Ratio of Males: Females	41:59	37:57	χ^2^ = 0.05, *p* = 0.816
Patients≤65 years of age	90 (90%)	91 (96%)	χ^2^ = 3.59, *p* = 0.058
Marital status	41 (41%)	15 (16%)	χ^2^ = 14.79, ***p*** **< 0.001**
Unemployed/Disability sick leave	74 (74%)	62 (66%)	χ^2^ = 1.49, *p* = 0.221
Age of first major depressive episode (Mean ± SD)	37.7 ± 15.3	26.4 ± 9.8	*t* = 6.14, ***p*** **< 0.001**
Early onset of major depressive episodes(< 25)	29 (29%)	52 (55%)	χ^2^ = 13.80, ***p*** **< 0.001**
Age of first psychiatric consultation (Mean ± SD)	35.9 ± 15.2	24.9 ± 10.7	*t* = 5.83, ***p*** **< 0.001**
Age of first psychiatric hospitalization (Mean ± SD)	40.1 ± 15.0	29.4 ± 11.7	*t* = 4.15, ***p*** **< 0.001**
Patients with recurrent depression (> 3)	39 (39%)	47 (50%)	χ^2^ = 2.37, *p* = 0.123
Duration of illness-current episode (years) (Mean ± SD)	11.9 ± 11.5	15.4 ± 12.1	*t* = −2.08, ***p*** **= 0.039**
Patients with comorbid substance use	27 (27%)	32 (34%)	χ^2^ = 1.14, *p* = 0.287
History of Alcohol use	9 (9%)	16 (17%)	χ^2^ = 2.77, *p* = 0.096
History of Cannabis use	6 (6%)	19 (20%)	χ^2^ = 8.71, ***p*** **= 0.003**
History of Cocaine use	3 (3%)	3 (3%)	χ^2^ = 0.006, *p* = 0.939
Number of Failed pharmacotherapies (Mean ± SD)	3.6 ± 2.6	5.3 ± 2.7	*t* = −4.31, ***p*** **< 0.001**
Failed antidepressant trials (Mean ± SD)	2.25 ± 1.8	1.63 ± 1.4	*t* = 2.63, ***p*** **= 0.009**
Failed SGA (Mean ± SD)	0.65 ± 0.7	1.68 ± 1.2	*t* = −7.17, ***p*** **< 0.001**
Failed Mood stabilizers (Mean ± SD)	0.3 ± 0.6	1.3 ± 0.9	*t* = −8.55, ***p*** **< 0.001**
Patients currently having psychotherapy	41 (41%)	78 (83%)	χ^2^ = 36.01, ***p*** **< 0.001**
Number of hospitalizations for depression since 1st episode			
None	56 (56%)	15 (16%)	χ^2^ = 33.48, ***p*** **< 0.001**
> one	20 (20%)	60 (64%)	χ^2^ = 38.41, ***p*** **< 0.001**
Patients with 1st degree relative with affective illness	51 (51%)	63 (67%)	χ^2^ = 5.13, ***p*** **= 0.023**
Patients with Anxiety disorders	61 (61%)	22 (23%)	χ^2^ = 27.97, ***p*** **< 0.001**
Patients with Panic disorders	19 (19%)	3 (3%)	χ^2^ = 12.04, ***p*** **< 0.001**
Patients with Melancholic symptoms	74 (74%)	40 (42%)	χ^2^ = 19.77, ***p*** **< 0**.**001**
Patients with Atypical symptoms	14 (14%)	25 (26%)	χ^2^ = 4.78, ***p*** **= 0.029**
Patients with suicidal attempts	23 (23%)	42 (44%)	χ^2^ = 10.22, ***p*** **= 0.001**
History of MDE with psychotic symptoms	27 (24%)	59 (63%)	χ^2^ = 25.11, ***p*** **< 0.001**
Patients with Personality disorders (Axis II in DSM-IV)	44 (44%)	45 (48%)	χ^2^ = 0.29, *p* = 0.589
Cluster A	7 (7%)	1 (1%)	χ^2^ = 4.31, ***p*** **= 0.038**
Cluster B	19 (19%)	16(17%)	χ^2^ = 0.128, *p* = 0.720
Cluster C	25 (25%)	5 (5%)	χ^2^ = 14.36, ***p*** **< 0.001**
Patients with Medical condition potentially relevant to treatment (Axis III in DSM-IV)			
Autoimmune diseases	11 (11%)	2 (2%)	χ^2^ = 6.101, ***p*** **= 0.014**
Cardiovascular diseases	25 (25%)	18 (19%)	χ^2^ = 0.962, *p* = 0.327
Chronic pain disorders	25 (25%)	15 (16%)	χ^2^ = 2.421, *p* = 0.120
Neurological conditions	13 (13%)	5 (5%)	χ^2^ = 3.396, *p* = 0.065
Metabolic disorder	10 (10%)	15 (16%)	χ^2^ = 1.532, *p* = 0.216

*SD* Standard Deviation, *TRD-UP* Treatment-Resistant Unipolar Depression, *BP* Bipolar disorder type I and type II, *MDE* Major depressive episode, *SGA* Second Generation Antipsychotics. Boldface indicates significant difference at an alpha level = 0.05

Patients with TRD-UP were significantly older than BP patients (46.5 ± 13.3 vs 40.6 ± 14.3, *p* = 0.003) whereas the two groups were equally distributed in terms of gender, with a female to male ratio close to 1.5.

The prevalence of patients who were married at the time of evaluation was significantly greater in the TRD-UP group compared to the BP patients (41% vs 16%, respectively; *p* < 0.001). Similar rates of unemployment or disability were noted in the two groups (74% TRD-UP vs 66% BP, *p* = 0.272).

### Clinical features and co-morbidities

BP patients had an early onset of MDE compared to TRD-UP patients (26.4 ± 9.8 vs 37.7 ± 15.3; *p* < 0.001). In line with this finding, BP patients had their first psychiatric consultation and their first psychiatric hospitalization at a younger age than TRD-UP patients (*p* < 0.001).

No differences were found between TRD-UP and BP concerning the prevalence of patients having recurrent depression (> 3 episodes) as well as the presence of comorbid substance use. However, BP patients had a higher prevalence of a history of cannabis use than TRD-UP (20% vs 6%, *p* = 0.005). The duration of the current episode of major depression was longer in BP than in TRD-UP (15.4 ± 12.1 vs 11.9 ± 11.5, *p* = 0.039).

Patients with TRD-UP showed a lower failure to different pharmacotherapies than BP patients (*p* < 0.001). Looking at the different pharmacological classes of psychotropic drugs, TRD-UP patients failed a greater number of antidepressant trials (*p* = 0.009) and a lower number of SGA (p < 0.001) and MS (p < 0.001) trials compared to BP patients.

Interestingly, the percentage of patients currently undergoing psychotherapy was significantly lower in TRD-UP than in BP patients (*p* < 0.001).

The prevalence of patients who did not have any hospitalization for depression since the first episode was greater in the TRD-UP group than in the BP group (p < 0.001). In contrast, BP patients showed greater prevalence of more than one hospitalization for depression since the first episode than TRD-UP (p < 0.001). Family history was also another characteristic that differed among the two groups. BP patients showed a higher prevalence of having at least one first-degree relative with affective disorders than TRD-UP patients (*p* = 0.029).

Of note, the prevalence of patients who had a history of suicidality was significantly higher in those affected by BP than those diagnosed with TRD-UP (*p* = 0.002). TRD-UP patients displayed higher prevalence of anxiety (*p* < 0.001) and panic (*p* < 0.01) disorders as well as depression with melancholic features (p < 0.001) than BP patients.

### Personality disorders and medical conditions

Overall there was no difference in the prevalence of personality disorders (Axis II in DSM-IV-TR) and physical diseases (Axis III in DSM-IV-TR) between the TRD-UP and BP; however, when studying the individual clusters, TRD-UP patients had a significantly higher prevalence rate of Cluster C personality disorders (avoidant, dependent and obsessive compulsive personality) compared to BP patients (*p* < 0.001). No differences were found for Clusters A and B personality disorders.

With the exception of autoimmune diseases that were more prevalent in TRD-UP than in BP patients, there were no differences on other Axis III co-morbidities.

### Depression severity and functioning

Using different psychometric scales, we examined and compared the severity of depression (MADRS, HAMD-17, QIDS-C16 and CGI-S) and the global functioning (GAF score) between TRD-UP and BP disorders. As shown in Table 2, TRD-UP patients were move severely depressed than BP patients as indicated by higher scores on MADRS, HAMD-17, QIDS-C16 and CGI-S scales (p < 0.001). In contrast, the global functioning of BP patients was higher than that of TRD-UP patients (p < 0.001).

**Table 2:** Severity of depression and global functioning of patients with TRD-UP and BP. Data are reported as Mean ± SD

Depression severity	TRD-UP (n = 100)	BP (n = 94)	Statistics
MADRS	30.8 ± 8.95	23.2 ± 6.98	*t* = 6.66, ***p*** **< 0.001**
HAMD-17	23.2 ± 6.24	17.0 ± 4.61	*t* = 7.92, ***p*** **< 0.001**
QIDS	15.8 ± 4.26	12.7 ± 3.91	*t* = 5.31, ***p*** **< 0.001**
CGI-S	5.0 ± 1.21	4.3 ± 1.18	*t* = 4.19, ***p*** **< 0.001**
GAF	55.5 ± 10.22	60.9 ± 4.66	*t* = −4.93, ***p*** **< 0.001**

*TRD-UP* Treatment-Resistant Unipolar Depression, *BP* Bipolar disorder type I and type II, *MADRS* Montgomery–Asberg Depression Rating Scale, *CGI-S* Clinical Global Impression-Severity of Illness, *QIDS* Quick Inventory of Depressive Symptomatology, *HAM-D17* Hamilton-Rating Scale for Depression, *GAF* Global Assessment of functioning, *SD* Standard Deviation. Boldface indicates significant difference at an alpha level = 0.05

### Predicting categorical diagnosis

We used binary logistic regression to evaluate which demographic and clinical characteristics were differently associated with TRD-UP or BP (Table 3). The binary logistic regression model consisted of 11 variables involving individual characteristics (i.e., age of first depression, marital status, psychotherapy, employment status, more than one hospitalizations, first degree relatives with affective disorders), presence of comorbidities (anxiety disorders), clinical features of depressive episode (i.e., HAMD-17 score, melancholic features, number of failed pharmacotherapies) and level of overall functioning (GAF score). We excluded from the model, panic and Cluster C personality disorders since very few individuals (*n* ≤ 5) were affected by these disorders in at least one of the two groups.

**Table 3:** Logistic regression showing odd ratios associated with TRD-UP instead of BP disorder (*N* = 194)

Variables	Coefficient B	SEM	Wald	*P* value	OR Exp(B)	OR 95% CI	
						Lower	Upper
Individual characteristics							
Age of 1st depression	−0.051	0.025	4.003	0.045	0.951	0.905	0.999
Marital status	1.583	0.701	5.108	0.024	4.872	1.234	19.235
First degree relatives with affective disorders	0.262	0.593	0.195	0.659	1.299	0.406	4.157
More than one hospitalization for depression	−2.148	0.614	12.239	**0.001**	0.177	0.035	0.389
Psychotherapy treatment	−2.232	0.699	10.190	**0.001**	0.107	0.027	0.423
History of suicide attempt	−0.135	0.625	0.047	0.829	0.874	0.257	2.974
Comorbidities							
Current anxiety disorders	2.357	0.608	15.033	**0.001**	10.560	3.208	34.763
Severity of depression							
Melancholic depressive features	0.650	0.747	0.755	0.385	1.915	0.422	8.285
Failed pharmacotherapies	0.266	0.106	6.331	0.012	1.304	1.060	1.604
HAM-D17 score	−0.193	0.067	8.208	**0.004**	0.824	0.722	0.941
Level of functioning							
Global assessment of functioning score (GAF)	0.164	0.047	11.985	**0.001**	1.178	1.074	1.293

*TRD-UP* Treatment-Resistant Unipolar Depression, *BP* Bipolar disorder type I and type II, *OR* Odds ratio, *CI* Confidence interval, *SEM* Standard Error of Mean. Boldface indicates significant association at an alpha level = 0.01

Our classification analysis reflected an overall goodness of fit to the data (χ^2^ = 168.8 *p* < 0.001 df = 11). Nagelkerke’s (0.775) indicated a moderately strong relationship between predictors and variable grouping. No multicollinearity between the variables was detected (VIF range: 1.137–1.725).

A combination of five variables (more than one hospitalization for depression, comorbidity with anxiety disorders, current psychotherapy, severity of depression (HAM-D17 score) and global functioning (GAF score) was able to significantly differentiate patients with TRD-UP from those with BP (Table 3). Of note, patients who were in psychotherapy and who had more than one hospitalization for depression had respectively 82% and 89% increased likelihood to have BP instead of TRD-UP. In contrast, the presence of a comorbidity with anxiety disorders increased by 10 times the likelihood of having a diagnosis of TRD-UP than of BP. Moreover, a lower depression severity as measured by the HAMD-17 score as well as a higher global functioning as measured by the GAF score increased the likelihood of having a diagnosis of BP instead of TRD-UP.

### Sociodemographic and clinical characteristics in BP-I, BP-II and TRD-UP

As secondary aim of this study we examined the possible differences within the bipolar spectrum (BP-I vs. BP-II) and then towards TRD-UP. As reported in Table 4, we found that some sociodemographic and clinical characteristics differentiated BP-I from BP-II patients and either BP-I or BP-II from TRD-UP. BP-I but not BP-II patients were younger than TRD-UP patients (*p* = 0.011). Patients with BP-II showed a lower rate of unemployment/sick leave than TRD-UP and BP-I patients (48% vs 74% and 80%, respectively; *p* < 0.001).

**Table 4:** Socio-demographic and clinical characteristics of patients with TRD-UP, BP-I and BP-II (*N* = 194)

	TRD-UP (*n* = 100)	BP-I (*n* = 52)	BP-II (*n* = 42)	Statistics
Age (Years) (Mean ± SD)	46.5 ± 13.3	39.7 ± 15.2^+^	41.7 ± 13.2	*F*_2,191_ = 4.6, *p* = 0.011
Ratio of Males: Females	41:59	21:31	16:26	χ^2^ = 0.11, *p* = 0.949
Patients≤65 years of age	90 (90%)	49 (94%)	42 (100%)	χ^2^ = 4.83, *p* = 0.089
Marital status	41 (41%)	8 (15%) ***	7 (17%) ***	χ^2^ = 14.81, *p* = 0.001
Unemployed/Disability sick leave	74 (74%)	42 (80%)	20 (48%)* **,^###^	χ^2^ = 13.67, *p* = 0.001
Age of first major depressive episode (Mean ± SD)	37.7 ± 15.3	28.9 ± 10.1^+++^	23.2 ± 8.6^+++^	*F*_2,191_ = 21.0, *p* < 0.001
Early onset of major depressive episodes(< 25)	29 (29%)	30 (58%) ***	22 (53%) ***	χ^2^ = 14.07, *p* = 0.001
Age of first psychiatric consultation (Mean ± SD)	35.9 ± 15.2	24.2 ± 10.5^+++^	25.9 ± 11.2^+++^	*F*_2,191_ = 16.8, *p* < 0.001
Age of first psychiatric hospitalization (Mean ± SD)	40.1 ± 15.0	28.1 ± 11.7^+++^	31.3 ± 12.6^+^	*F*_2,123_ = 10.0, *p* < 0.001
Patients with recurrent depression (> 3)	39 (39%)	27 (52%)	20 (48%)	χ^2^ = 2.55, *p* = 0.279
Duration of illness-current episode (years) (Mean ± SD)	11.2 ± 10.4	12.2 ± 11.6	16.7 ± 12.2	*F*_2,191_ = 2.6, *p =* 0.075
Patients with comorbid substance use	27 (27%)	20 (39%)	12 (29%)	χ^2^ = 2.21, *p* = 0.331
History of Alcohol use	9 (9%)	6 (12%)	10 (24%)	χ^2^ = 5.89, *p* = 0.053
History of Cannabis use	6 (6%)	11 (21%)	8 (19%)	χ^2^ = 8.81, *p* = 0.012
History of Cocaine use	3 (3%)	1 (2%)	2 (5%)	χ^2^ = 0.63, *p* = 0.730
Number of Failed pharmacotherapies (Mean ± SD)	3.6 ± 2.6	5.0 ± 2.5^++^	5.6 ± 2.8^+++^	*F*_2,191_ = 9.9, *p* < 0.001
Failed antidepressant trials (Mean ± SD)	2.5 ± 1.7	1.35 ± 1.4^++^	2.0 ± 1.4	*F*_2,191_ = 5.4, *p* = 0.005
Failed SGA (Mean ± SD)	0.65 ± 0.7	1.9 ± 1.1^+++^	1.4 ± 1.2^+++^	*F*_2,191_ = 30.2, *p* < 0.001
Failed Mood stabilizers (Mean ± SD)	0.3 ± 0.6	1.3 ± 0.8^+++^	1.4 ± 1.0^+++^	*F*_2,191_ = 37.5, *p* < 0.001
Patients currently having psychotherapy	41 (41%)	43 (83%) ***	35 (83%) ***	χ^2^ = 36.01, *p* < 0.001
Number of hospitalizations for depression since 1st episode				
None	56 (56%)	1 (2%) ***	14 (33%) ***, ^###^	χ^2^ = 43.36, *p* < 0.001
> one	20 (20%)	39 (75%) ***	21 (50%) ***	χ^2^ = 44.41, *p* < 0.001
Patients with 1st degree relative with affective illness	51 (51%)	31 (60%)	32 (76%)*	χ^2^ = 7.76, *p* = 0.021
Patients with Anxiety disorders	61 (61%)	9 (17%) ***	13 (31%) ***	χ^2^ = 29.74, *p* < 0.001
Patients with Panic disorders	19 (19%)	2 (4%) **	1 (2%) **	χ^2^ = 12.09, *p* = 0.002
Patients with Melancholic symptoms	74 (74%)	22 (42%) ***	18 (42%) ***	χ^2^ = 19.77, *p* < 0.001
Patients with Atypical symptoms	14 (14%)	11 (21%)	14 (33%)*	χ^2^ = 6.93, *p* = 0.031
Patients with suicidal attempts	23 (23%)	17 (33%)	25 (60%) ***,^##^	χ^2^ = 17.73, *p* < 0.001
History of MDE with psychotic symptoms	24 (24%)	16 (31%)	4 (10%)	χ^2^ = 6.18, *p* = 0.045
Patients with Personality disorders (Axis II in DSM-IV)	44 (44%)	26 (50%)	19 (45%)	χ^2^ = 0.50, *p* = 0.777
Cluster A	7 (7%)	0 (0%)	1 (2%)	χ^2^ = 4.65, *p* = 0.098
Cluster B	19 (19%)	6 (12%)	10 (24%)	χ^2^ = 2.49, *p* = 0.287
Cluster C	25 (25%)	1 (2%) ***	4 (10%) ***	χ^2^ = 15.38, *p* < 0.001
Patients with Medical condition potentially relevant to treatment (Axis III in DSM-IV)				
Autoimmune diseases	11 (11%)	2 (4%)	0 (0%)	χ^2^ = 6.65, *p* = 0.036
Cardiovascular diseases	25 (25%)	10 (20%)	8 (19%)	χ^2^ = 0.96, *p* = 0.618
Chronic pain disorders	25 (25%)	8 (15%)	7 (17%)	χ^2^ = 2.44, *p* = 0.295
Neurological conditions	13 (13%)	3 (6%)	2 (5%)	χ^2^ = 3.42, *p* = 0.181
Metabolic disorder	19 (19%)	20 (38%)*	12 (29%)	χ^2^ = 6.83, *p* = 0.033
Depression severity (Mean ± SD)				
MADRS	30.8 ± 8.9	22.2 ± 6.7^+++^	24.3 ± 7.2^+++^	*F*_2,191_ = 22.7, *p* < 0.001
HAMD-17	23.2 ± 6.2	16.7 ± 4.7^+++^	17.3 ± 4.5^+++^	*F*_2,191_ = 30.8, *p* < 0.001
QIDS	15.8 ± 4.2	12.1 ± 3.7^+++^	13.4 ± 4.0**	*F*_2,191_ = 15.4, *p* < 0.001
CGI-S	5.0 ± 1.2	4.2 ± 1.3^++^	4.3 ± 1.1^++^	*F*_2,191_ = 8.8, *p* < 0.001
GAF	55.5 ± 10.2	60.9 ± 4.4^+++^	60.3 ± 4.9^++^	*F*_2,191_ = 11.7, *p* < 0.001

*SD* Standard Deviation, *TRD-UP* Treatment-Resistant Unipolar Depression, *BP-I* Bipolar disorder type I, *BP-II* Bipolar Disorder type II, *MDE* Major depressive episode, *SGA* Second Generation Antipsychotics

*p < 0.05, **p < 0.01, ***p < 0.001 versus UP-TRD; ^#^*p* < 0.05, ^##^*p* < 0.01, ^###^*p* < 0.001 BP-I versus BP-II by Pearson’s chi-square test

^+^*p*< 0.05, ^++^*p* < 0.01, ^+++^*p* < 0.001 versus UP-TRD by One-way Anova plus Bonferroni post hoc analysis

The prevalence of patients who did not have any hospitalization for depression since the first episode was greater in BP-II than in BP-I (p < 0.001). Family history was also another characteristic that differed amongst groups. BP-II patients showed a higher prevalence of having at least one first-degree relative with affective disorders than TRD-UP patients (*p* = 0.010).

Of note, the prevalence of patients who had a history of suicidality was significantly higher in those affected by BP-II than those diagnosed with TRD-UP (p < 0.001) and BP-I (*p* < 0.01). We did not observed difference between BP-I and BP-II patients concerning the prevalence of comorbid substance use, of anxiety disorders, of Axis II personality disorders, and Axis III physical diseases. In addition, no difference was found between BP-I and BP-II for the levels of both depression severity and global functioning.

## Discussion

These results indicate that patients with TRD-UP exhibit different psychopathological features compared to depressive episodes in patients with BP, suggesting that TRD-UP is a distinct psychopathological condition and not a prodromal state of BP depression.

TRD-UP patients show higher depression severity, higher prevalence of anxiety and panic disorders and of Cluster C personality disorders, a later onset of depression and fewer hospitalizations than BP patients. Within the bipolar spectrum, BP-II patients show lower rate of unemployment and hospitalizations for depression and higher prevalence of history of suicide attempts than BP-I patients.

Using a binary logistic regression model, it was possible to distinguish TRD-UP from BP disorders. The following variables were mostly associated with TRD-UP than with BP: increased anxiety, lower score on the GAF scale, higher depression symptoms (HAMD-17 score), lower number of hospitalizations and psychotherapies.

Overall, these findings are in agreement with previous literature comparing major depressive disorder (MDD) (non-TRD) with BP [15, 36, 37]. Indeed, the higher depression severity in TRD-UP than in BP was also reported in previous studies differentiating BP from MDD (non-TRD) patients. Additionally, earlier onset of depression, a greater prevalence of family history of affective disorders and a higher rate of suicide attempts were found in BP compared with UP (non-TRD) depression [15, 36, 37].

Mitchell and Malhi [37], in an extensive review, described a higher prevalence of depressive episodes and lower functioning in BP compared with UP (non-TRD) depression. In our study, TRD-UP showed the same number of recurrent episodes but higher number of hospitalizations and a lower GAF score compared with BP, pointing out the severity of the TRD-UP condition in comparison with UP (non-TRD) and BP. The lower functioning in TRD-UP patients is in line with previous studies indicating that TRD-UP, unlike BP patients, tend to experience more unremitting depressive states and higher fluctuations with depressive symptoms despite receiving appropriate treatment [38]. Patients with BP depression showed a greater prevalence of atypical symptoms and lower prevalence of melancholic features than TRD-UP patients as previously indicated by Benazzi [39]. However, when accounting for possible confounding variables, in the binary logistic model, depression with atypical or melancholic features was not significantly associated with TRD-UP, as suggested in a previous study [40].

In TRD-UP, we found a greater prevalence of Cluster C personality disorders in keeping with Kornstein and Schneider [41] and a meta-analysis reporting that patients with affective disorders had more than 50% comorbidity with personality disorders [42].

We have shown that BP patients had a greater prevalence of metabolic disorder comorbidity compared with the TRD-UP group. These findings are in line with some studies where lifetime comorbidity in BP-I patients were reported to be between 50% up to 70% [43]. However, it cannot be ruled out that the higher rate of metabolic disease observed in BP patients was caused by the higher use of SGA in BP than in TRD-UP patients (as described in the methodology section).

Finally, patients with TRD-UP have less number of failed pharmacological trials compared to BP, especially for SGA and MS. This might be due to the polypharmacy required in BP versus TRD-UP, as previously mentioned in a youth population at risk for BP disorders [44].

Altogether, these findings suggest that TRD-UP may constitute a unique subtype of depression compared with other affective disorders, and thus depressive episodes in BP are different than those in TRD-UP. Moreover, they are in support of a bi-dimensional approach for TRD-UP and BP disorders, recognizing points of differentiation that might contribute to distinguish a diagnosis within the affective disorders. Of note, it seems that differences also exist between TRD-UP and the different sub-types of BP. Further studies with a larger sample size may allow to deeply examine the psychopathological features that may be specifically associated with either TRD-UP, BP-I or BP-II. These results could seem in apparent contrast with Angst et al. [45] arguing that a diagnostic change from depression to BP-I and BP II occurs in about 1% and 0.5% of patients per year, respectively, and supporting a spectrum theory, in which UP depression and BP depression are in a continuum spectrum [46].

In our study, we have chosen a priori patients followed in the Mood Disorders clinic for at least 2 years (mean 7.5 years), in which the possible risk of novel manic/hypomanic episode and thus consequent change in diagnosis was minimized and ruled out. This is also in agreement with a recent systematic review and meta-analysis underscoring that the rate of conversion from UP to BP disorders decreases with time reaching 0.8% in 10 years of initial diagnosis [47]. For this reason, compared to the systematic review by Bennabi et al. [16] and Dudek et al. [15], bipolarity was not a risk factor for TRD. However, in keeping with Bennabi et al. [16], comorbidity for anxiety disorders was a clear risk factors for TRD-UP.

In contrast with Cassano et al. [48] and Benazzi [23], we have not used scales such as the Structured Clinical Interview for the Mood Spectrum or the Hypomania interview guide that by characterizing threshold and subthreshold mood episodes, hypomanic or “temperamental” features related to mood dysregulation allow assessing hypomanic symptoms. This limitation has prevented us to detect if TRD-UP could also present sub-threshold hypomanic symptoms. Another limitation of our study is that this is a retrospective and observational cross-sectional chart-review analysis that consequently lacks randomization and a longitudinal follow up. Prospective longitudinal studies are warranted to demonstrate that TRD will not convert in BP depression, or at least in a non-significant extent.

Despite the above-mentioned limitations, this study has several strengths: this is the first comparison study examining different clinical and sociodemographic data from an outpatient tertiary clinic for affective disorders proposing different predictors to distinguish TRD-UP from BP depression. Moreover, it adds clinical evidence towards the differentiation of TRD-UP as a unique type of depression as previously hypothesized by Fagiolini and Kupfer [49] suggesting that TRD-UP may have specific clinical characteristics, neurobiological profile, and environment in which TRD develops, requiring a combination of AD and SGA as a first-line treatment [17].

Therefore, our study supports the ancient hypothesis of K. Schneider differentiating endogenous periodic unipolar depression (a chronic condition with several episodes in lifespan, and resistant to treatment) from bipolar phasic depression (characterized by phases of mania and depression) and exogenous depression (caused by exterior factors, with less episodes during life) [50, 51].

However, to fully validate Schneider’s hypothesis, these results should be replicated with larger controlled studies and include a comparison group with UP depressive patients who are not treatment resistant.

Finally, further analysis of longitudinal studies addressing neurobiological markers, clinical features between the TRD-UP and BP disorders should provide insight concerning these particular questions and evaluate the implications on pharmacological outcomes. This integrated approach will aid clinicians and researchers to disentangle initial diagnostic controversies between unipolar and bipolar spectrum improving the differential management and therapeutics of patients suffering from depression.

## Conclusion

This retrospective and observational cross-sectional study shows that patients with depressive episodes in TRD-UP have a different history and distinct psychopathological features compared with BP depressive patients, thus TRD-UP constitutes a distinct psychopathological condition and not necessarily a prodromal state of BP depression. Further studies are needed to differentiate the pharmacological responses and outcomes in these distinct groups.

## Abbreviations

AD: Antidepressant
ADHD: Attention Deficit-Hyperactivity disorder
ANOVA: Analysis of variance
BP: Bipolar disorder
BP-I: Bipolar Type I disorder
BP-II: Bipolar Type II disorder
CGI-S: Clinical Global Impression-Severity of Illness
DSM-IV-TR: Diagnostic and Statistical Manual of Mental Disorders, Fourth Edition. Text revision
GAF: Global assessment of functioning score
HAM-D17: Hamilton-Rating Scale for Depression
MADRS: Montgomery–Asberg Depression Rating Scale
MDC: Mood Disorders Clinic
MDD: Major depressive disorder
MDE: Major depressive episode
MS: Mood stabilizers
MSM: Maudsley Staging Method
MUHC: McGill University Health Center
QIDS-C16: Quick Inventory of Depressive Symptomatology
SCID: Structured Clinical Interview for Diagnosis
SD: Standard deviation
SEM: Standard error of the mean
SGA: Second-generation antipsychotics
STAR*D: Sequenced Treatment Alternatives to Relieve Depression
TRD-UP: Treatment-Resistant Unipolar Depression
YMRS: Young Mania Rating Scale
